# Slim V-shaped frenectomy: Description of a minimally invasive surgical technique

**DOI:** 10.34172/japid.025.3524

**Published:** 2025-04-22

**Authors:** Abdusalam E. Alrmali, Paolo Nava, Jacob Martin Zimmer, Syed Hanan Rufai, Andreas Onisiforou, Hom-Lay Wang

**Affiliations:** ^1^Department of Periodontics and Oral Medicine, University of Michigan School of Dentistry, Ann Arbor, MI, USA; ^2^Department of Oral Medicine, Oral Pathology, Oral and Maxillofacial Surgery, University of Tripoli School of Dentistry, Tripoli, Libya; ^3^Department of Biologic and Materials Sciences & Prosthodontics, University of Michigan School of Dentistry, Ann Arbor, MI, USA

**Keywords:** Dental, Esthetics, Labial frenulum, Minimally invasive surgical procedures, Oral frenectomy, Periodontics

## Abstract

This article introduces a minimally invasive slim V-shaped surgical technique for labial frenectomy, specifically targeting high labial frenulum attachment. Proper frenulum management is critical in dental specialties, influencing aesthetics, phonetics, and prosthetic rehabilitation outcomes. The described technique is adaptable to various types of labial frenulum, including mucosal, gingival, papillary, and papilla penetrating. It involves a slim V-shaped incision, consecutive frenulum detachment, and repositioning within the vestibule, effectively addressing potential recurrence growth. Its minimally invasive nature reduces wound expansion into neighboring structures, ensuring optimal healing and minimizing postoperative discomfort. In conclusion, the slim V-shaped surgical technique offers a promising solution, minimizing complications and maximizing treatment success for high labial frenulum attachment.

## Introduction

 Proper frenulum management is pivotal in treating patients across many dental specialties. Frenula are described as small folds of tissue that connect various structures within the oral cavity, present at different locations within the oral cavity. These are the maxillary labial frenum, the mandibular labial frenum, and the lingual frenulum. Their primary function is to stabilize the upper and lower lip and the tongue.^1^ Histologically, these structures comprise muscular and connective fibers externally enveloped by a layer of oral mucosa.^2^

 The labial frenulum is located at the maxillary midline towards the vestibulum. There is a significant degree of variability regarding its level of frenulum insertion.^3^ In most cases, the labial frenulum inserts on the vestibular aspect of the alveolar process near the junction of keratinized gingiva and alveolar mucosa.^4^ Mirko et al^5^ classified labial frenal attachments into four categories: mucosal attachment, wherein the fibers are attached up to the mucogingival junction; gingival attachment, when the fibers are inserted within the attached gingiva; papillary attachment, where the fibers extend into the interdental papilla; and papilla penetrating, where the frenal fibers cross the alveolar process and extend up to the palatine papilla.

 Abnormal frena are associated with several genetic and chromosomal conditions such as Ehlers-Danlos syndrome (EDS), infantile hypertrophic pyloric stenosis (IHPS), Ellis-van Creveld syndrome (EVCS), and more.^1^ Moreover, high labial frenulum attachment can negatively affect aesthetics and movement of the upper lip. Diastema, gingival recession, and impairment of the patient’s phonetics and oral hygiene practices have been reported in high labial frenulum attachment cases.^6,7^

 Thus, labial frenectomy is part of orthodontic, prosthetic, or periodontal treatment planning.^8^ Various techniques have been proposed for oral frenulum removal, but most of the described techniques are more invasive.^9^ In contrast, the described technique effectively preserves the keratinized gingiva, essential for maintaining periodontal health. This method ensures that the gingival tissues remain largely intact by strategically repositioning the frenulum rather than completely removing it. The minimally invasive approach to labial frenectomy, described by Kadkhodazadeh et al,^10^ significantly reduces scarring, promoting faster healing and improved aesthetic outcomes. The study describes a minimally invasive technique for labial frenectomy that achieves superior aesthetic and functional outcomes, along with minimal postoperative discomfort and a low recurrence rate.

## Technique description

**Figure 1:**
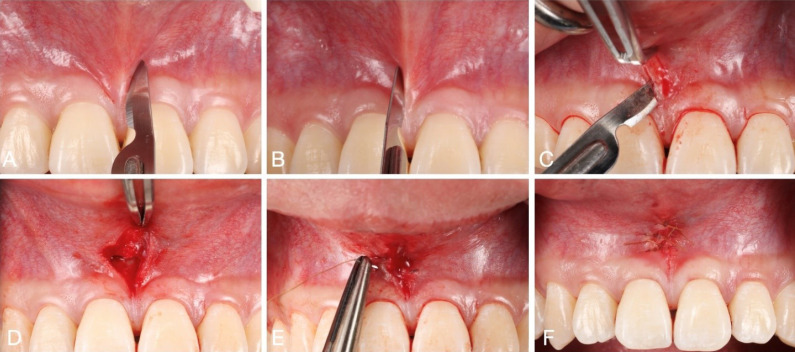
(A and B) Incision design in the shape of a slim V around the frenulum with #15c blade. (C) Horizontal incision to remove frenulum attachment from its underlying periosteum. (D) The mucosa is repositioned apically towards the vestibule. (E) Suturing with single interrupted resorbable 5-0 chromic gut sutures. (F) Final suture after Slim V-shaped frenectomy

**Figure 2:**
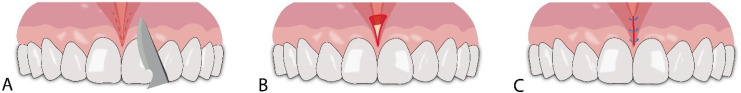
Surgical steps for the in slim V-shaped technique. (A) Incision design: A slim V-shaped incision is made around the frenulum using a #15c blade. (B) Repositioning of mucosa: The residual mucosa, along with the frenulum fibers, is repositioned apically towards the vestibule, with a small area of bone exposed to ensure proper movement of the frenum attachment apically. (C) Suturing: Single interrupted resorbable sutures are placed to secure the repositioned tissue

 The minimally invasive slim V-shaped surgical technique for labial frenectomy described here is indicated for treating high labial frenulum attachment of mucosal, gingival, papillary, and papilla-penetrating types. The surgical site is anesthetized through vestibular infiltration targeting the frenulum area. It is advised to use an anesthetic agent with a vasoconstrictor to manage excessive bleeding from the frenulum area. Two nearly parallel incisions, at a 45-degree angle to the bone shaped like a slim V, are made, starting from the tip of the coronal frenulum and extending to the apical frenulum attachment within the vestibulum (Figures 1 and 2). The incisions are placed close to the frenulum, encompassing its attachment. This will minimize the area that needs to heal by secondary intention, subsequently reducing scar formation and improving the zone of keratinized gingiva. The authors recommend using a #15 or #15c blade for incisions. The frenulum attachment is detached from the underlying periosteum with a cut almost parallel to the bone. The frenulum is then excised, and the residual mucosa with the frenum fibers is repositioned apically within the vestibulum. Complete detachment and removal of the frenulum from the periosteum, followed by apical suturing, are crucial to prevent its reattachment and regrowth. The site is closed with simple interrupted sutures, starting at the apical part (key suture) and passing through the mucosa and periosteum to approximate the wound edges within the oral mucosa (Figures 1 and 2). A 5-0 resorbable chromic gut suture is used to prevent ingrowth within the mucosa. The patients are advised to follow postoperative instructions, including a soft diet and daily oral rinses with warm salt water to aid healing. Complete epithelial healing of the surgical site is typically observed two weeks postoperatively.

**Figure 3:**
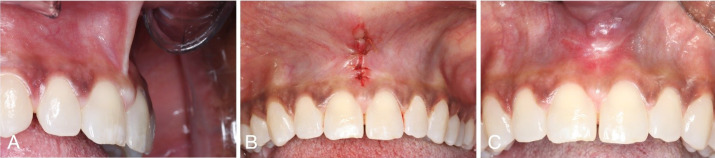
(A) Initial presentation with high labial frenulum attachment. (B) Final suture after slim V-shaped frenectomy. (C) Situation at the 2-week postoperative follow-up

**Figure 4:**
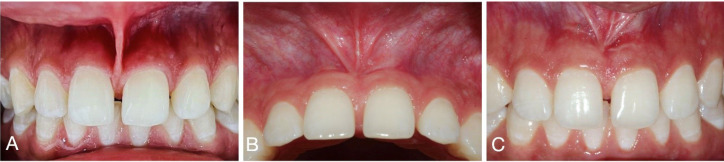
(A) Initial presentation with high labial frenulum attachment with papilla penetrating type. (B) 3 months follow-up after slim V-shaped frenectomy. (C) 6 months follow-up after slim V-shaped frenectomy

 Successful clinical short- and long-term outcomes of the slim V-shaped frenectomy can be seen in Figures 3 and 4. Figure 3 shows the initial presentation, suturing, and 2-week postoperative visit after proceeding with the slim V-shaped frenectomy for high labial frenulum attachment. The patient initially presented with high labial frenulum attachment of gingival type. To accommodate for the high frenulum attachment, the frenulum was removed with the minimal invasive slim V-shaped frenectomy. The patient reported no postoperative pain or discomfort. Two weeks after the procedure, appropriate healing of the surgical site could be observed (Figure 3). Figure 4 shows more long-term outcomes of the technique.

 The patient presented with high labial frenulum attachment before planned orthodontic treatment for midline diastema. Clinical examination showed high labial frenulum attachment of papillary penetrating type and the presence of maxillary diastema at sites #8 and #9. To accommodate for the high frenulum attachment, slim V-shaped frenectomy was proceeded. Figure 4 shows successful surgical outcomes at 3 and 6 months.

## Discussion

 High labial frenulum attachment has been linked to impaired lip movement, diastema, gingival recession, and difficulties maintaining adequate oral hygiene.^11,12^ Moreover, high frenulum pull has been reported to negatively influence treatment outcomes of soft and hard tissue grafting procedures.^8,13^ This emphasizes the importance of successful frenectomy with adequate techniques.

 Multiple surgical techniques for frenectomy have been described in the literature, including the classic frenectomy according to Archer and Kruger, Miller’s technique, Z-plasty, and V-Y plasty. Techniques like Z-plasty and the Miller frenectomy do not address papillary involvement. In a traditional frenectomy, part of the papilla often must be removed if the frenum has invaded it, leading to significant esthetic issues for the patient.^14^ Alternatively, electrosurgery or lasers can be used instead of a scalpel blade. In general, sufficient removal of the frenulum is crucial to achieve treatment success and prevent recurrent growth of the frenulum. However, there has been a growing emphasis on preserving the papilla in the esthetic zone in recent years, as regenerating lost papilla is challenging, if not impossible.^10^ Alternative to a scalpel blade, electrosurgery or lasers can be utilized. In general, sufficient removal of the frenulum is crucial to achieve treatment success and prevent recurrent growth of the frenulum. Extending the incisions into neighboring structures, such as deep into the vestibule or the attached gingiva, can make the wound site disproportionately large. This can delay wound healing, which occurs by secondary intention, often resulting in severe scar formation that might lead to the shallowing of the vestibule.^15^ Additionally, it can result in increased postoperative morbidity and a prolonged recovery period. These factors must be carefully considered, especially in patients with a high smile line, since the site is located within the esthetic area. The slim V-shaped frenectomy described here addresses these considerations effectively. Nevertheless, potential postoperative complications should be considered. These may include mild swelling, bleeding, and transient discomfort, which are common in the immediate postoperative period but typically resolve quickly with proper care. Additionally, patients are advised to adhere to a soft diet and maintain oral hygiene with gentle rinses to facilitate recovery. The recovery period for this minimally invasive technique is generally shorter compared to more traditional methods.

 By limiting wound extension into neighboring structures, the slim V-shaped frenectomy promotes faster healing, reduces scar formation, and minimizes postoperative morbidity. In the mesiodistal direction, the V-shaped incisions proceed close to the base of the frenulum. Therefore, the area that will heal by secondary intention within the attached gingiva can be minimized. In the apical direction, the incisions are limited to the frenulum area from tip to base and not disproportionally extended within the vestibulum. The technique stresses the importance of completely removing the frenulum attachment to the underlying periosteum and apical positioning of the remaining mucosa. This is crucial to prevent regrowth of the frenulum into the surgical site. Precisely placed simple interrupted sutures to correctly position and approximate the wound margins will further optimize proper wound healing. If executed accordingly, minimal postoperative patient discomfort and scarring can be expected from this technique. It must be emphasized that the technique described here is intended for labial frenectomy, not lingual frenectomy. However, it is suitable for treating high labial frenulum attachments of mucosal, gingival, papillary, and papilla-penetrating types. One limitation of this technique is that it may not be applicable, particularly for complex anatomical variations or recurrent scar frenulum.

## Conclusion

 In conclusion, we introduced an innovative, minimally invasive surgical technique for labial frenectomy. The meticulous application of the described steps in this approach not only minimizes postoperative complications but also enhances the potential for maximizing treatment success.

## Competing Interests

 The authors declare that they have no competing interests.

## Consent for Publication

 Written informed consent was obtained from all participants for both study procedures and publication of the collected data.

## Data Availability Statement

 The data regarding this study would be provided upon reasonable request from the first author.

## Ethical Approval

 None.
